# Integrated Care for Older People with Different Frailty Levels: A Qualitative Study of Local Implementation of a National Policy in Luton, England

**DOI:** 10.5334/ijic.6537

**Published:** 2023-03-23

**Authors:** Nimra Khan, Gurch Randhawa, David Hewson

**Affiliations:** 1 Institute for Health Research University of Bedfordshire Department of Psychiatry University of Oxford, UK; 2 Institute for Health Research University of Bedfordshire, UK

**Keywords:** frailty, integrated care, older people with different frailty levels, England

## Abstract

**Introduction:**

The NHS England General Medical Services 2017–18 contract made it mandatory for general practices in England to identify and manage older people proactively. In response to the national policy, the Luton Framework for Frailty (LFF) programme was developed to target older residents of Luton and offer interventions according to their frailty level. The aim of this study was to gain a deeper understanding of the LFF and the factors that affect the implementation of a proactive integrated care service for older people with different frailty levels (OPDFL).

**Methods:**

We undertook document analyses and conducted semi-structured interviews with stakeholders to create a ‘thick description’ that provides insights into the LFF.

**Results:**

Healthy ageing interventions bring beneficial outcomes but to increase the uptake they should be co-produced with older people. A common electronic system within primary care and multidisciplinary team meetings (MDT) aid implementation. However, variation in implementation across Luton, different levels of buy-in for MDT, and different data systems in primary and secondary care make implementation challenging.

**Conclusion:**

The LFF is a promising initiative and lessons learned are likely to be transferable to other settings as proactive management of frailty takes on greater policy prominence in the UK and worldwide.

## Introduction

Frailty is a condition related to the ageing process in which multiple body systems gradually lose their in-built reserves [1,2,3]. Frailty is associated with negative outcomes such as falls and fractures, nursing home admissions, ambulance calls and emergency department visits, hospital admissions and death [1]. Accordingly, frailty poses a huge burden on individuals and their families, as well as on health and care systems with already limited resources [4]. The prevalence of mild, moderate and severe frailty among older people in the UK aged ≥65 years is estimated to be 35%, 12% and 3%, respectively [4]. Frailty poses a burden to an already economically challenged and demand-led National Health Service (NHS) in the UK.

The NHS long term plan published in 2019 recognised that existing services are not responsive to the needs of older people with frailty [5]. This plan aimed to help people age well and stay in their own home independently for longer. It set out three new service models to improve the care for older people with frailty. These included improving NHS care in care homes, identifying and providing proactive care to older people with frailty who are community dwelling, and finally to provide rapid community responses during crises. One of the initiatives to improve the support for older people with frailty living in care homes was the Enhanced Health in Care homes. This programme aimed to align each care home with a general practice by 2020, with care home residents having access to emergency out of hours support and receiving medications reviews from pharmacists. The NHS long term plan also emphasised that services in the community would be made more responsive so that older people do not have unwanted hospital visits during crisis situations. To achieve some of these goals, structural changes have also been introduced in the NHS to make it more integrated, such as the development of Primary Care Networks (PCN). General practices have joined together as groups of practices to work with other services such as community, mental health, social care, pharmacy, hospital, and voluntary services in their local areas to ensure provision of proactive, personalised and coordinated care. The introduction of integrated care systems in 2022 is another initiative for developing partnerships between organisations to plan and deliver health and care services locally to improve outcomes [6]. Clinical Commissioning Groups (CCG) and PCNs also have to implement the anticipatory care models by 2023/2024 to provide proactive care for people who are at high risk of adverse outcomes, for example those with frailty or multimorbidity. These individuals should be identified using screening tools such as the electronic Frailty Index (eFI) [7] and their care planning should be done by a multidisciplinary team. The primary outcome for this initiative is to add five extra years of healthy life.

One policy approach that has been proposed to reduce the costs of frailty management is to focus efforts on prevention, proactive care and earlier intervention [8]. This requires primary care providers to plan and manage care for older adults with frailty and to ensure integration and continuity of this care. The first step undertaken by the NHS to implement this new policy approach was the requirement in the General Medical Service (GMS) contract in 2017/2018 in England for general practices to identify and optimally manage all older people aged ≥65 years with moderate or severe frailty. The long-term goal is to make frailty assessment and management an integral part of primary care practice. General Practitioners (GPs) are required to identify their more complex patients and proactively organize high quality care in collaboration with other services, with this scheme aiming to improve outcomes for older people and reduce the burden on acute care services [8].

**Figure 1:**
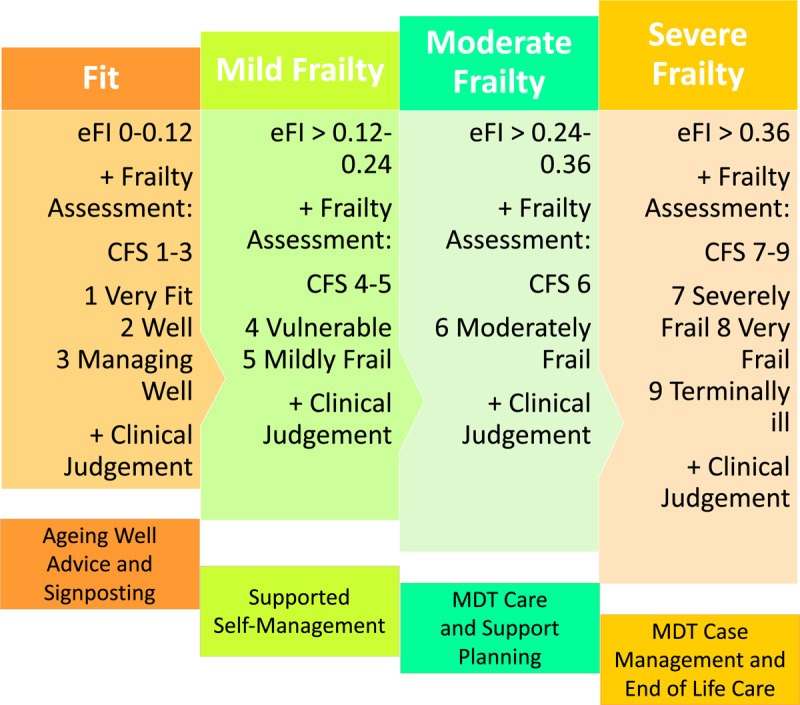
Luton Framework for Frailty Programme.

According to this programme, all general practices should identify older people ≥65 years who have frailty using the eFI [7]. The eFI uses information routinely recorded in a person’s electronic health record to calculate a frailty score based on the presence or absence of 36 health deficits, following the accumulation of deficits model of frailty [8]. The eFI, which has been made available to all general practices, assigns older people into one of four categories, which are fit, mildly frail, moderately frail or severely frail. The policy emphasises that people should be assigned a frailty category based, not only on the eFI score, but also on clinical assessment using a valid tool such as the Clinical Frailty Scale (CFS) [9] or on clinical judgement [5]. The Luton Clinical Commissioning Group (Luton CCG) has used these policy recommendations to develop the Luton Framework for Frailty (LFF) Programme, which is shown in Figure 1.

Luton is a town situated in the southeast of England with a population of 225,300, of which 11.7% people are aged over 65 years (10). Luton is an ethnically diverse town with nearly 55% of the population from Black, Asian and Minority Ethnic (BAME) groups [10]. Luton is the 59th most deprived area out of the 326 local authorities in England [11], and is in the 20% of most deprived unitary authorities with major health inequalities across the socioeconomic gradient. Among those residents identified as least deprived, life expectancy is 10.4 and 6.3 years higher for men and women, respectively [12]. Primary care in Luton is carried out by 26 GP practices, which are organised into five Primary Care Networks.

The LFF is an integrated programme, which is offered to older people aged ≥65 years who are residents of Luton. Using the eFI score and the general practitioners’ clinical judgement, an individual is assigned one of the four categories mentioned previously, with services offered according to their frailty level. For example, those people who are classified as fit receive an annual leaflet providing information on promoting healthy ageing, while those people who have mild frailty are offered an opportunity to participate in a free 12-week physical activity programme called the “Healthy Ageing Programme”. Those people who are identified as having either moderate or severe frailty are provided with an individually tailored case management intervention. Although the LFF was first introduced in October 2018, no comprehensive evaluation has been conducted yet to determine how it has been implemented or if the outcomes described in Box 1 have been achieved. The aim of this study was to gain a deeper understanding of the LFF and the factors that affect the implementation of an integrated care programme for older people with different frailty levels (OPDFL).

**Box 1 Information on the Luton Framework for Frailty Programme: Model of Care**

**Older People: Over 65 years**

The main aim of this programme is to promote healthy ageing, to case find frail elderly, proactively manage their care and reduce the need for older people, those aged over 65, to be urgently admitted to hospital.

This will be achieved through system-wide agreement, development and implementation of **Luton Framework for Frailty Programme**; clearly describing the interventions and services across health & social care that will **support older people with healthy ageing to remain in their own home for as long as possible**.

Where this is no longer possible, it **ensures that the best possible care is provided for older people in residential & nursing settings**.

The framework describes the offer for each frailty cohort; fit, mild, moderate and severe.

**Improving Care for Older People, those over 65, in Luton:** 

**What are the desired outcomes?**

✔ Older people are supported with healthy ageing and to remain in their own home for as long as possible. Where this is no longer possible, it ensures that the best possible care is provided for older people in residential & nursing settings.
✔ Older people will experience improved health and care that focuses on ‘what matters most to them’.
✔ Older people will experience less need to be urgently admitted to hospital.
✔ Older people will experience less use of unnecessary medicines.
✔ Older people will be supported to stay stable, strong & safe.
✔ Older people’s chances of a ‘first fall’ being injurious is reduced.
✔ Older people will experience effective treatment of injurious falls, helping them return to maximum independence.
✔ Older people who are recurrent fallers have their well-being maximised.
✔ Older people will have the chance to discuss their wishes and preferences.
✔ Older people will be cared for and die in their preferred place.

Outcome measures will include quality of life, the number of hospital admissions, rate of institutionalisation, health and social care expenditure, and mortality.

## Methods

### Study Design

A qualitative approach using thick description was used in this study. A thick description is a qualitative method to study social practices such as the delivery of care services within their specific contexts [13]. Different research techniques are used to provide an in-depth explanation of a social practice, such as a care delivery programme. Thick description begins by using documentary analysis to describe hard facts about the programme, however, these alone are not enough to provide an understanding of what happens when the programme is implemented. This requires soft facts, based on questions like how and why, which are obtained from qualitative interviews with stakeholders. The information gathered from the documentary analysis and the interviews is taken together to provide an in-depth understanding of the programme [14,15]. When drafting this manuscript we adhered to the COnsolidated criteria for REporting Qualitative research (COREQ) [16].

### Data collection procedure

We studied a variety of documents about the programme: official documents related to the programme, presentations given by project leaders, meeting minutes, factsheets about the LFF, the business case, documents regarding the strategic priorities and objectives of the Luton CCG, and documents about specific working groups related to the care programme. Most documents were provided by the project leaders of the LFF.

Purposive sampling was used to invite 22 stakeholders from primary care, community and acute services in Luton, with 18 semi-structured interviews conducted to collect data. The participants included GPs, pharmacists, programme leads, managers, commissioners and geriatricians. The job descriptions of the participants are presented in Table 1. The interview questions were developed to reflect the aim of the study, which is to understand the programme provided to OPDFL in Luton. Interviews with service providers started by asking them about services provided by their organisation for OPDFL, the referral mechanisms they use, as well as the information and communication mechanism they use and any implementation issues they had faced. The interviews were expected to last for 25–40 minutes. Due to the COVID-19 pandemic, face-to-face interviews were not possible, so a range of communication systems were used according to the preference of the person being interviewed (Skype, Zoom, MS Teams, and telephone). By interviewing stakeholders from diverse backgrounds, different perspectives about the programme were gained.

**Table 1:** Job description of the participants.


PARTICIPANTS	JOB DESCRIPTION

**P1**	Senior Leadership Role

**P2**	Senior GP

**P3**	Senior Manager of a Service

**P4**	Senior Manager of a Service

**P5**	Pharmacist

**P6**	Senior Manager of a Service

**P7**	Senior Geriatrician

**P8**	Team Lead for a Service

**P9**	Team Lead for a Service

**P10**	Senior Leadership Role

**P11**	Senior Pharmacist

**P12**	Pharmacist

**P13**	Commissioner

**P14**	Senior Commissioner

**P15**	GP

**P16**	Senior GP

**P17**	GP

**P18**	Senior GP


### Data Analysis

Information gathered through the document review was structured according to the LFF. The first author conducted the interviews and discussed the notes made with the other two authors. Documents review and interviews were analysed by the first and second authors, the second author reviewed a sample of the documents and interviews, and the findings were discussed within the team. The analysis was done using Mayring’s content analysis method [14]. Both deductive and inductive coding were used as the topics were determined a priori but any new themes emerging were also considered. For each aspect of the LFF, sentences were selected that helped explain the current practices. The information in this manuscript describes the main elements that characterise the LFF and offers a deeper understanding of the factors that affect the implementation of an integrated care service for older people with different frailty levels (OPDFL).

### Ethical Consideration

Ethics approval was obtained from the Institute for Health Research Ethics Committee (IHREC), University of Bedfordshire (IHREC950).

## Results

**Figure 2:**
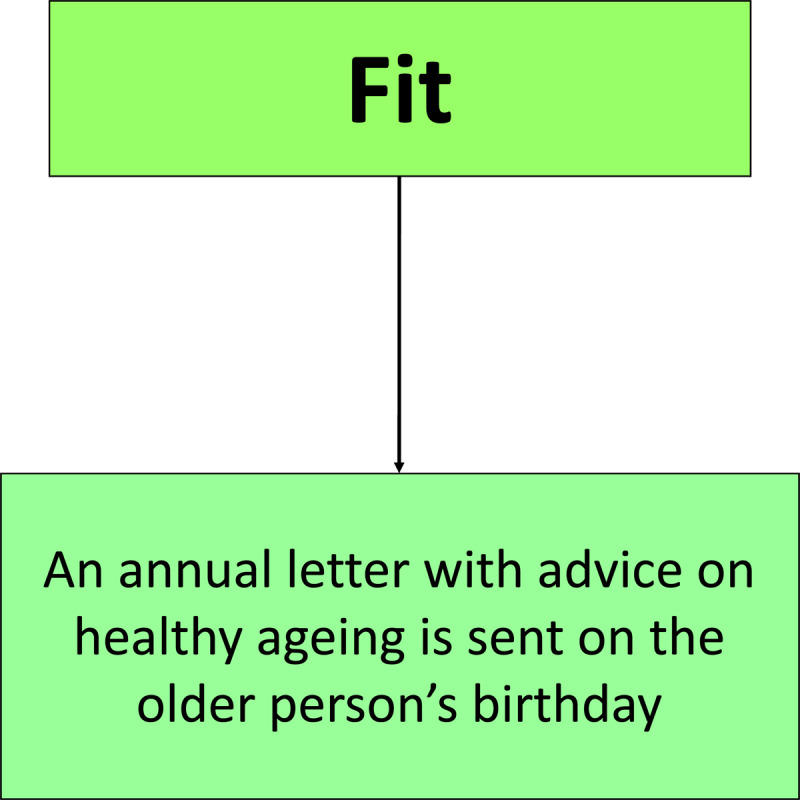
The care pathway for older people who are considered to be fit.

Based on the documentary analysis and semi-structured interviews conducted with stakeholders, we developed diagrams showing the care pathways for OPDFL (Figures 2, 4 and 5). The implementation of the LFF, which started in October 2018, aimed to promote healthy ageing, case find older people with frailty, proactively manage their care, and reduce the need for older people to be urgently admitted to hospital. The LFF starts by case finding OPDFL and then offers them different care pathways. Below is a description of the programme and insights into the factors that affect the implementation of the care pathways.

### Case Finding

At the general practice level, case finding for frailty is done by running the eFI score on the electronic patient records. Older people who have an eFI score of 0–0.10 and a CFS score of 1–3 are considered to be fit; those with an eFI score of 0.13–0.24 and CFS score of 4–5 are considered to have mild frailty; those with an eFI score of 0.25–0.36 and CFS score of 6 are considered to have moderate frailty and those with an eFI score > 0.36 and CFS score of 7–9 are considered to have severe frailty. Based on an individual’s frailty level, different services are offered, and people have different care pathways.

### Care Pathway – Fit

Older people who are considered to be fit receive an annual letter about healthy ageing from their GP on and after their 65^th^ birthday. They can access community-based services such as Active Luton physical activity classes offered by the local leisure centres. Furthermore, if they need support in terms of advice for health and social care services, befriending and socialising service or help in household tasks, they can access a voluntary organisation called Age Concern Luton. The care pathway for older people who are considered to be fit was rolled out across Luton (Figure 2).

### Care Pathway – Mild Frailty

Older people considered to have mild frailty are offered the opportunity to take part in a free-of-charge 12-week Healthy Ageing Programme (HAP). This programme delivers strength and balance training to older people (>65 years old) with mild frailty who are residents of Luton, with the aim of decreasing the risk of falls in this group. The programme started in March 2019 with an initial pilot using three general practices. During the pilot phase, 13 different physical activity offers were included, covering activities such as Yoga, Pilates, Gentle Exercise, Boxercise and Dancing. The programme was subsequently scaled-up to include all general practices in 2020 during the COVID-19 pandemic delivered in an online format.

**Figure 3:**
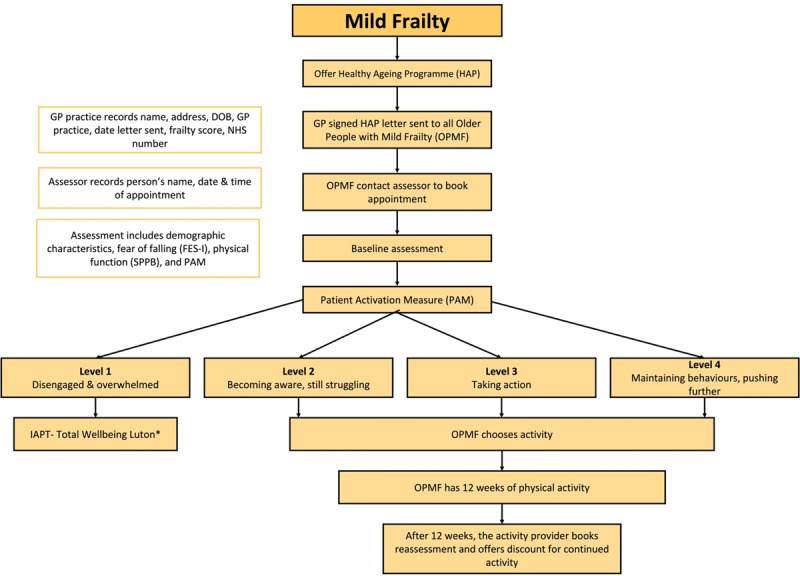
Care Pathway for Older People with Mild Frailty.

The general practices identify older people over the age of 65 years with mild frailty and who are residents of Luton. They receive letters with details of the HAP and are signposted to contact assessors if they are interested in taking up the offer. The assessors work for a Community Wellbeing Trust, Active Luton, which has been commissioned by the Luton CCG. The role of the assessors is to collect data on demographic and outcome indicators at baseline, record attendance of people who sign up for HAP and collect data on outcome indicators at the end of the programme. Those who contact the assessors are given an appointment to meet the assessors in person. On the day of the appointment, people are given an explanation about the different exercise programmes in which they can participate. If they agree to participate, tests for measuring physical functioning, activation level and fear of falling are performed using the short physical performance battery (SPPB), patient activation measure (PAM) and falls efficacy scale (FES), respectively [17,18,19]. The participants can then undertake 12 weeks of physical activity free of charge. At the end of the 12 weeks, participants are re-assessed for SPPB, PAM and FES and given offers to continue the exercise classes for 12 weeks at low prices. The pathway is shown in Figure 3.

#### Factors affecting the implementation of the care pathways for fit and mild frailty

An initial evaluation of the HAP pilot conducted in 2019 (described above) showed improvement in outcomes for older people with mild frailty for instance, there were statistically significant and clinically relevant improvements in the SPPB and PAM scores and a decrease in the fear of falling. The complete evaluation of the HAP pilot will be published separately. The number of participants who accepted the offer was high but adherence to the interventions offered was low. Four hundred people signed up for the programme but data on the attendance and follow-up was only collected from 55 people. The number of participants who withdrew were not recorded by the assessors or by the organisations delivering the interventions. Therefore, it was not clear if the health assessors did not collect data on attendance and follow-up, or if people withdrew from the programme. Nearly 50% of the population of Luton is from ethnic minority groups [20], however the participation from minority groups was very low. Furthermore, participation from the non-minority, English was also low. The interviews with stakeholders offered some reasons for the low adherence rate, with language thought to be a greater potential barrier than the type of exercises offered, as the offers included some culturally appropriate options. Another reason for the low uptake was that the exercises offered were considered to be too easy by some participants, while transport was also an issue for some people who wanted services to be nearby, rather than having to travel by car or public transport to the venue.

Furthermore, it was thought that despite having some interventions for older people who are fit and who have mild frailty, system-wide there has always been a focus on those who are severely frail and the need to reduce hospital admissions and institutionalisation and less focus on quality of life and preventative activities for those who are fit or only mildly frail.

*“We are not getting a lot of people from the ethnic minority group but actually we have got white population who are not responding either. And I have spoken with people who do the assessment, they say a lot of exercises we offer seem too easy so we offer chair based exercise and the participants feel they don’t need chair based exercise, instead they need a more physical exercise … We need to offer services at door steps instead of people getting on bus to go somewhere and change to another bus” (P13, Commissioner)*

*“The lower number of participants from ethnic minority groups doesn’t appear to be a cultural issue as a variety of culturally appropriate activities e.g. yoga in the Hindu temple are offered. However, some participants have attended the assessment with sons/daughters to act as interpreters. So, language barrier might be an issue” (P10, Senior Leadership Role)*

*“I think continuing challenge is that we focus too much on those who are struggling at the end so those who’ve got complexity and frailty and we too often forget to do the preventative work for those who are functioning well …we focus on prevention of hospital admissions, prevention of admissions to care homes when in fact what we should really be focusing on is people, what is their quality of life…” (P2, Senior GP)*

### Care Pathway – Moderate or Severe Frailty

**Figure 4:**
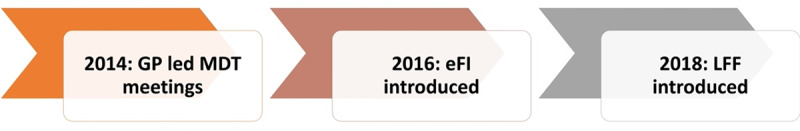
Timeline of the services introduced for older people with different frailty levels in Luton.

**Figure 5:**
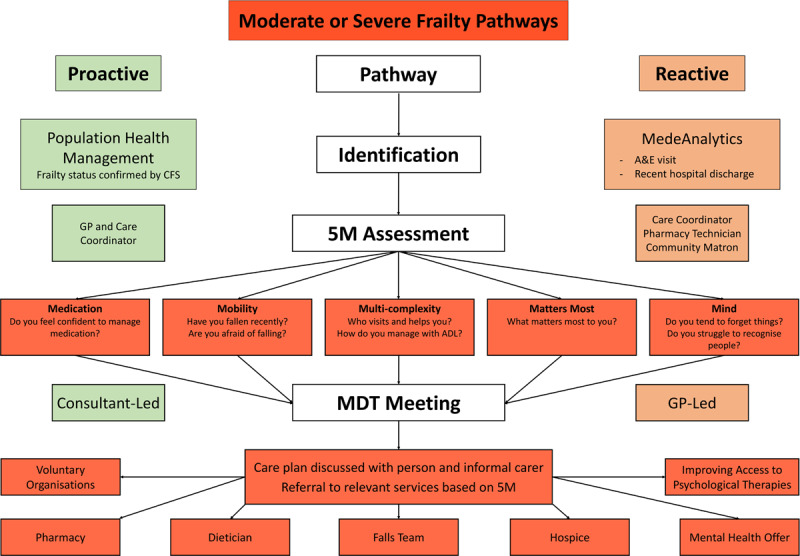
Proactive and reactive care pathways for older people with moderate or severe frailty.

Older people with moderate or severe frailty already had services in place prior to the LFF but after the identification and management of frailty became the requirement in GMS contract in 2017/18, new pathways were introduced in addition to the existing pathways. A timeline has been included to show when the different pathways were introduced (Figure 4). There were GP led MDT meetings, which have been conducted since 2014. Complex patients were identified by the GP and discussed monthly by the GP, community matron and care coordinator. In 2016, the eFI was introduced to identify older people with moderate and severe frailty to discuss in the GP led MDT meetings. In 2017/18, LFF was introduced, and two pathways for older people with moderate and severe frailty were introduced: Proactive pathway and reactive pathway, as shown in Figure 5.

These pathways had some new components, and some existing services which were merged with these new services, for example the GP led MDT meeting of the reactive pathway were already there since 2014. Both pathways were expected to be implemented across Luton.

#### Proactive Pathway

The proactive pathway is initiated by identifying older people with moderate or severe frailty through the health-risk management tool on a monthly basis by a GP and care coordinator. The health risk tool was developed by one of the organisations involved in providing services to older people with moderate and severe frailty. It has information on eFI score, demographics, health conditions a person was suffering from it, hospital episodes or A & E visits. Running this health risk tool helps identify population with higher risk of adverse outcomes. Those identified to be at risk of adverse outcomes are phoned by the care coordinator who conducts a 5M assessment (mobility, matters most, mind, medication, multi-complexity) and those who are considered as having higher risk of deterioration are discussed in a consultant led MDT meeting [21]. The consultant led MDT meetings occur weekly, and include a senior GP, falls prevention team, specialist nurses such as respiratory nurses, dietician, palliative care team, care coordinator and a consultant from the Department of Medicine for the Elderly at the hospital. This MDT meeting has less representation from social care team, who are mostly present in acute care pathways (not in the remits of this study). The MDT discussed cases from across Luton and were not for specified practices.

A care plan is developed during the MDT meeting, which is discussed with the older person and their carers, and the person is referred onto other services. Those identified with complex needs and at increased risk of deterioration are offered intensive case management, which involves the community matron adding these individuals in their case load and tagging them as red. They are then referred to other services such as the respiratory team, adult social care, physiotherapy, and are given equipment (if needed), then followed up by the community matron until their condition improves, whereby the community matron tags them green and takes them off the case load.

The care plans developed in all the MDT meetings (proactive and reactive pathways) use a widely used electronic template, which contains elements of anticipatory care and covers DNAR (do not attempt resuscitation) decisions. The electronic template also includes wider treatment escalation plans, such as whether someone prefers admission to hospital, community care with active treatment, or end of life care in the community with symptom palliation.

A key role is played by the care coordinator who has several responsibilities. They liaise with GP surgeries, facilitate MDT meetings in the GP surgeries, ensure attendance of different professionals, send out agendas and minute meetings, follow up on actions decided in the meeting, enter information into the patient medical record, contact people at high risk, and conduct the 5M assessment. Care coordinators do not require any specific qualifications; however, they are very experienced and should have knowledge of all the services available for older people or their carer.

During the COVID-19 pandemic, the proactive pathway was stopped.

#### Reactive Pathway

Older people whose health is deteriorating and who have had either an A&E visit or who have been recently discharged from hospital are identified through a MedeAnalytics database (MedeAnalytics Inc, Richardson, TX, USA) on a daily basis by a team consisting of a care coordinator, pharmacy technician and community matron. The care coordinator conducts a 5M assessment via phone, with those who are considered as having a higher risk of deterioration are discussed in the GP led MDT meetings (described above) which occurs monthly or bi-monthly (based on the number of older people in the catchment area). After the COVID-19 pandemic, the reactive pathway continued.

For those whose conditions escalate, there is a Rapid Response team, which provides urgent care, within 2 hours between 8 am and 8 pm, 365 days a year, to patients in their own homes or residential homes, with the aim to avoid unnecessary conveyance and admission to hospital. They receive referrals from professionals such as GPs, paramedics, 111, the hospital and residential homes. They also respond to calls from existing patients on community nursing caseloads with urgent unplanned palliative or catheter needs.

Post-COVID the Government announced that people who are clinically extremely vulnerable should be shielding. In Luton, older people with moderate and severe frailty were considered to be clinically extremely vulnerable and were told to shield. Professionals from GP practice used to make regular calls to this cohort to offer support.

There is a single data system known as SystmOne (The Phoenix Partnership, Leeds, UK) for most primary care providers, with care plans written on it that can be accessed by different providers. However, few providers have access to a read only mode for SystmOne in hospital, meaning that most of the providers based in hospital are not able to access a patient’s primary care records.

##### Factors affecting the implementation of the care pathways for moderate and severe frailty

Participants felt that having multi-professional inputs through the MDT have been beneficial to manage older people with complex conditions. They described the MDT as a good platform to connect with GPs as they are usually not easily accessible.

*“The MDTs are of great value I feel because ….having those different professionals around in the room… We don’t always have the same ideas about something and somebody might come with something that is completely different from your idea. I think that’s the real benefit of the MDT meeting” (P8, Team Lead for a Service)*

*“Sometimes even though you shouldn’t wait but getting hold of GPs is really difficult. It is quite a good forum for you to know that you have got a GP there” (P6, Senior Manager of a Service)*

*“So the benefit of the MDT is that rather than just a GP see a patient and trying to refer them everywhere you’ve got a wider team talking about what the patient’s needs are and you get a better perspective on how to manage those needs and some people like a dementia nurse or community matron might have a completely different perspective on how to care for the patient than just the GP and so you’ve got more skills wrapped around the patient..” (P16, Senior GP)*

Mostly stakeholders found MDTs useful, but they mentioned that their uptake is not the same across Luton. While some GPs talked about the benefits of the GP led MDT meetings, others mentioned that they are not even aware of when the MDTs occur. They felt that the only time they get some information about the MDT meetings is when a hospital discharge occurs, which suggests that there is poor communication in the system. Some other factors highlighted for variable uptake of GP led MDTs among practices were need for coordination, to facilitate attendance and build relationships with GPs that create an awareness that participating in MDTs can help them share care, reduce their work load and improve outcomes for older people with frailty.

*“Because I think (name of a GP) loves these MDT meetings and thinks it has a lot of value…but other GP surgeries are like I haven’t got time, it is an hour, we have got other things that we should be doing. There is different buy in from different surgeries. (P9, Team Lead for a Service)*

*“I’m not entirely sure they are set up properly to be honest I think the only time we get any news of MDTs is for those who have been discharged from the hospital and then they are discussed somewhere else…Well one of the big things is that I don’t know how and when they work. So I think it’s probably lack of information coming through into GP practices that may well be happening but I don’t know when are they happening or what was discussed…” (P18, Senior GP)*

*“The uptake of practice based frailty MDTs is poor because of a combination of factors such as improvement needed in coordinating a process, facilitating attendance and developing relationships such that GPs see that the work of caring for frailty patients can be shared and so actually reduces their workload and improves patient care” (P16, Senior GP)*

The interviews were conducted during the COVID-19 pandemic, with all participants mentioning how COVID-19 had negatively impacted upon the LFF. For instance, one of the issues faced was the shift in focus of all the healthcare organisations towards managing the pandemic and all proactive work was halted. In particular, the proactive pathway for the older people with moderate and severe frailty was stopped.

*“Covid-19 has made everything reactive, although actually in the early days of Covid-19 because we can see that people might be at risk of becoming severely ill with Covid-19 there was quite a significant push to advanced care planning and treatment escalation plan for our care home patients and also for some of our very severe patients just to think through what would you want if you got Covid-19, would you want to be actively treated, would you want to be admitted to hospital, Covid-19 in general, has meant that things like routine reviews so the proactive work has been put on hold” (P2, Senior GP)*.

Another challenge faced during the MDT meetings conducted as part of both the proactive care pathway and for care homes (described later), was the lack of preparation and clarity of what should be expected of an MDT meeting, which resulted in the potential waste of professional time.

*“It all depends on how well prepared people are in an MDT so if you put a name on a list and then you go to an MDT and no one has done any preparation you might just be asking does anyone know about this patient and if no one knows about them or done any planning then you’re only sharing your ignorance” (P16, Senior GP)*

*“So if somebody is relatively new to how MDTs work, puts forward a patient where it actually only requires maybe one discipline or two disciplines to be working with the individual it feels like it is not a good use of the whole team”..(P2, Senior GP)*

*“They’re not useful if you’re not clear on what you’re hoping to achieve. I think people have MDTs they call it MDT but it’s not really MDT so again, I think, the standard which can be inconsistent, and then people don’t get value out of it…You go into some MDTs and people just put people on them just to fill the gap and fill the meeting time” (P4, Senior Manager of a Service)*

Not having access to SystmOne in the hospital was highlighted by a geriatrician as a factor which leads to the inability to deliver integrated care.

*“We have a couple of people in the hospital that can access system one. That’s my sole integration is a couple of people in the hospital that can access system one records, because they’ve got view only access. So the answer at the moment is we’re not integrated at all” (P7, Senior Geriatrician)*

### Older People in Care Homes

The NHS Long Term Plan 2019, as part of its Ageing Well Programme, committed to the roll out of an Enhanced Health in Care Homes (EHCH) initiative, whereby care homes will be aligned with general practices from 2020 onwards [22]. In Luton, there are a total of 25 care homes with around 930 residents. The alignment of the care homes with the general practices started in March 2020, with all the care homes aligned by October 2020. A GP-led weekly check is performed to see if there are any new needs of residents. while a monthly GP-led MDT meeting is performed to review all the residents of the aligned care home. During the weekly care home check in the COVID-19 period, six questions were asked:

1. Who has COVID-19?
2. Who has had a recent hospital discharge?
3. Is anyone having signs of deterioration?
4. Is anyone having Anorexia?
5. Is anyone having signs of confusion?
6. Does anyone require medication review?

Furthermore, the care home staff ranked their residents into three categories: red indicated very worried; amber indicated might be having some problems; and green indicated stable. Based on residents’ needs, reviews were conducted in priority order for red, amber, and then green.

#### Factors affecting the implementation of the care home MDT meetings

The stakeholder interviews revealed a range of issues with this element of the programme. There was a perception that, prior to the EHCH, care homes had not received the kind of support from general practices that they needed. However, after the introduction of the EHCH began, coordination between the care homes and the general practices improved, with issues for care home staff resolved proactively. Some of the initial challenges included were that care home staff were not well prepared for the MDT meetings and did not pick the right residents for discussion. In addition, care home residents sometimes resented having to leave their GP and get a new GP. Furthermore, although initially GPs were reluctant to take up EHCH because of time constraints and increased workload, after implementation some GPs reported that the EHCH system actually made their workload more efficient. Furthermore, unlike practice based MDT meetings, there is 100% uptake of MDTs and check-ins in the care homes. The reason for better compliance with care home based MDT meetings was thought to be them being part of the newly introduced primary care network contract.

*“Absolutely invaluable even GPs are seeing the benefits now because they have got these relationships with the care homes…and the GPs even stopped the number of visits they had to make due to covid…one issue initially was that you’ve got some patients who have had the same GP from 20 or 30 years and that GP practice isn’t aligned to that care home, and now they go into a care home which is aligned with a different GP practice so the patient would say that I have perfect care from my GP from 30 years and I’m not changing” (P13, Commissioner)*

*“I think before care homes were sort of isolated, they will struggle to access the GP, they’d have to be on phone all day, they often be waiting around, some specialist might come and see the resident but wouldn’t feedback and they won’t know what is going around. The communication was very poor, with weekly check-ins and monthly MDTS, the care homes know once a week they will get all the attention they need and allows them to organise their workload, allows them to make sure that nobody gets ignored and the residents get the attention they need in a timely manner” (P12, Pharmacist)*

*“I think the reason for 100% uptake of MDTs and check- in in the care homes is due to their being part of the PCN DES contract” (P16, Senior GP)*

## Discussion

In this study we systematically described and analysed an integrated care programme for OPDFL known as the Luton framework for frailty (LFF) programme.

The LFF case finds OPDFL and offer them different interventions based on their frailty levels. For example, those identified as fit are given letters with healthy ageing advice. Older people who have mild frailty are offered an opportunity to take part in a 12-week free-of-charge physical activity programme. Those who have moderate or severe frailty are offered care by multidisciplinary teams. The LFF has several factors in common with other integrated care programmes for older people with frailty, such as case finding, conducting assessment, developing a personalised care plan, having a multidisciplinary team, and conducting follow-ups. However, the LFF distinguishes itself in that it includes interventions not just for older people who are already moderately or severely frail, but also for older people who are fit or have mild frailty [23,24,25,26]. Furthermore, the LFF is a specific local implementation of a national policy.

It was found that there is a low uptake of physical activity interventions among older people with mild frailty, both from ethnic minority and non-minority populations. Some of the reasons described by stakeholders were that exercises were perceived to be easy by the older people; venues were not close to their homes [27]; language barriers for the ethnic minority population [28] and lastly, stakeholders felt that the focus has traditionally been on those who are already frail, with less offered for those who are considered as fit or having mild frailty [29].

The care pathways for those with moderate or severe frailty, MDTs were considered useful as they offered multi-professional inputs and easy access to GPs [30]. But their uptake was not the same [31] across Luton and sometimes attendees of the MDTs were not aware of what to expect from it leading to a waste of professional time.

This in-depth qualitative inquiry of the LFF included the perspectives of stakeholders from across the system including GPs, senior management, nurses, pharmacists and care coordinators. This has provided insights into the life of a programme in terms of how it evolves over time and what helps or becomes a challenge for implementation. We recruited a sample of stakeholders from across the system, however, we were only able to recruit one senior geriatrician, and therefore we could not get perspectives of professionals from acute care services regarding the LFF. Nevertheless, we used purposive sampling and were able to get a sample with stakeholders from diverse backgrounds.

Another limitation of this study is the lack of representation from the service users. We did not interview older people and their informal carers, although this was initially planned to gain the perspectives of older people and their carers about the interventions within the LFF. However, the time allocated for data collection for this study overlapped with the first two waves of COVID-19, and since older people were most negatively impacted by the pandemic [32,33,34,35,36], recruiting older people for this study became very difficult because the organisations in Luton who had to provide support in recruiting participants thought that older people were already being contacted by too many services and were overburdened, so should not be asked for interviews at this time. Instead, it was suggested that the researchers should wait for some months. This study was part of a PhD and had time limitations, therefore, qualitative interviews with older people were not conducted.

This is the first study to explore the implementation of a national frailty management policy at a local level and lessons learned could be used by others in similar contexts to design and improve their programmes.

We have studied an integrated care programme and its implementation. However, in our study we found that all the interventions offered for OPDFL had their unique challenges in implementation. Keeping those challenges in view, we recommend that future programmes offering physical activity for older people with mild frailty should design the intervention with inputs from the older people. Recording data is vital for evaluation for instance, in this study assessors did not capture the data on the number of people who were sent the HAP offer letters, people attending the exercise classes and those who withdrew from the intervention. This is a gap, as one cannot assess the uptake and adherence to the programme.

Stakeholders in Luton and in other similar contexts introducing care pathways which include MDT meetings should quantitatively measure uptake and adherence to the intervention and explore qualitatively views of those who do not adhere to the intervention. In this study the data on uptake and adherence to the care pathways among service providers were not available. Finally, as the NHS moves towards adopting integrated care services, there is need to have common information systems across primary and secondary care services, as not having common data systems is a huge barrier to delivering integrated care.

Lessons learnt from the implementation of programmes such as LFF are important to inform forthcoming policy initiatives, such as the anticipatory care model, which has to be implemented by the CCGs and PCNs by 2023/2024.

## Conclusion

This study presents findings of an in-depth qualitative analysis of programme for OPDFL and the factors which affects its implementation. The integrated LFF programme offered different interventions for OPDFL. Providing care pathways for older people who are fit or mildly frail are a good initiative but making them successful requires inputs from older people while designing the interventions. Furthermore, developing MDTs to manage older people who are moderate or severely frail is useful in managing complex cases, but there should also be an evaluation to understand why there is variation in its implementation. Lastly, there should be data systems that are common for both primary and secondary care.
